# Risk investigation of in-stent restenosis after initial implantation of intracoronary drug-eluting stent in patients with coronary heart disease

**DOI:** 10.3389/fcvm.2023.1117915

**Published:** 2023-03-10

**Authors:** Hongfei Xi, Jiasi Liu, Tao Xu, Zhe Li, Xuanting Mou, Yu Jin, Shudong Xia

**Affiliations:** ^1^ Department of Cardiology, the Fourth Affiliated Hospital, International Institutes of Medicine, Zhejiang University School of Medicine, Yiwu, China; ^2^ Department of Neurology, The Second Affiliated Hospital, Zhejiang University School of Medicine, Hangzhou, China

**Keywords:** drug-eluting stent, in-stent restenosis, coronary heart disease, nomogram, prediction model

## Abstract

**Objective:**

To analyze the risk factors of in-stent restenosis (ISR) after the first implantation of drug-eluting stent (DES) patients with coronary heart disease (CHD) and to establish a nomogram model to predict the risk of ISR.

**Methods:**

This study retrospectively analyzed the clinical data of patients with CHD who underwent DES treatment for the first time at the Fourth Affiliated Hospital of Zhejiang University School of Medicine from January 2016 to June 2020. Patients were divided into an ISR group and a non-ISR (N-ISR) group according to the results of coronary angiography. The least absolute shrinkage and selection operator (LASSO) regression analysis was performed on the clinical variables to screen out the characteristic variables. Then we constructed the nomogram prediction model using conditional multivariate logistic regression analysis combined with the clinical variables selected in the LASSO regression analysis. Finally, the decision curve analysis, clinical impact curve, area under the receiver operating characteristic curve, and calibration curve were used to evaluate the nomogram prediction model's clinical applicability, validity, discrimination, and consistency. And we double-validate the prediction model using ten-fold cross-validation and bootstrap validation.

**Results:**

In this study, hypertension, HbA1c, mean stent diameter, total stent length, thyroxine, and fibrinogen were all predictive factors for ISR. We successfully constructed a nomogram prediction model using these variables to quantify the risk of ISR. The AUC value of the nomogram prediction model was 0.806 (95%CI: 0.739–0.873), indicating that the model had a good discriminative ability for ISR. The high quality of the calibration curve of the model demonstrated the strong consistency of the model. Moreover, the DCA and CIC curve showed the model's high clinical applicability and effectiveness.

**Conclusions:**

Hypertension, HbA1c, mean stent diameter, total stent length, thyroxine, and fibrinogen are important predictors for ISR. The nomogram prediction model can better identify the high-risk population of ISR and provide practical decision-making information for the follow-up intervention in the high-risk population.

## Introduction

CHD is a chronic disease that seriously affects human life and health. Its morbidity and mortality are increasing every year (1). Percutaneous coronary intervention (PCI) is an essential treatment method for coronary artery disease (2).

ISR refers to the gradual restenosis of primary coronary artery lesions after stent implantation, defined as the diameter of coronary luminal stenosis ≥50% throughout the stent and/or within the proximal and distal 5 mm segments of the stent (3). And ISR usually develops six months after PCI and is characterized by recurrent angina pectoris, but myocardial infarction can also occur. In recent years, with the gradual replacement of bare metal stents (BMS) by DES, the incidence of ISR has been significantly reduced (4). However, ISR after DES implantation, with an incidence of 3%–20% (5), is still one of the main reasons affecting the long-term efficacy of PCI, and there is no standardized treatment strategy (6). Thus, the prevention and treatment of ISR remain a challenging problem in the cardiovascular field.

The exact mechanism of ISR is still not fully understood (4). Therefore, early detection and control of related risk factors may be an important means to reduce the incidence of ISR and improve patients' prognosis and quality of life. There still needs to be more suitable prediction models to predict ISR. However, several previous studies have analyzed potential predictors associated with high-risk ISR and developed predictive models based on them (5, 7, 8). Some limitations suppress their clinical application, such as poor stability and weak discriminative power of prediction models, and poor generalizability of predictors. In addition, we can find that most of the studies focus on the comprehensive search for predictors of ISR and do not fully consider the ease of use for clinicians (9, 10). Therefore, there is an urgent need to establish a reliable, accurate, and easy-to-use ISR prediction model for clinicians and patients to accurately predict the occurrence of ISR and make clinical decisions for primary prevention.

## Methods

This study retrospectively analyzed the clinical data of patients with CHD who received DES treatment for the first time in the Fourth Affiliated Hospital of Zhejiang University School of Medicine from January 1, 2016, to January 1, 2020. LASSO regression and multivariate logistic stepwise regression analyses were used to determine the risk factors of ISR. Based on these predictors, we constructed a nomogram prediction model to evaluate the risk of ISR in different patients quantitatively.

### Study population

We included 414 patients with CHD who were first treated with DES in our hospital between January 1, 2016, and June 1, 2020. The institutional review boards of the Fourth Affiliated Hospital of Zhejiang University School of Medicine approved this study.

The inclusion criteria were as follows: (i) patients with acute coronary syndromes; (ii) patients who received DES for the first time in our hospital; (iii) DES was performed according to the Chinese Guidelines for Percutaneous Coronary Intervention (2016); (iv) the presence of ISR was determined by coronary angiography during follow-up; (v) relevant demographic characteristics, laboratory and imaging data can be obtained from the hospital information system. And the exclusion criteria include (i) patients who received bare metal stent implantation; (ii) patients who underwent PCI outside the hospital during the follow-up period; (iii) patients who underwent coronary artery bypass grafting during the follow-up period; (iv) patients with previous heart failure, cardiomyopathy, or myocarditis; (v) patients with severe hepatic or renal insufficiency; (vi) patients with advanced malignant tumors.

### Data collection

All clinical data came from the information system of the Fourth Affiliated Hospital of Zhejiang University School of Medicine. The clinical data included demographic information, past medical history, medication history, coronary angiography characteristics, and laboratory and imaging findings at the first PCI. These data are the results of perioperative and follow-up examinations. All patients received a standardized PCI strategy. Moreover, they were treated with oral aspirin (100 mg/d) in combination with clopidogrel (75 mg/d) or ticagrelor (90 mg twice daily) for one year after emergency PCI and elective PCI. Patients typically have repeat coronary angiography approximately one year after PCI to determine whether ISR has occurred. And patients with postoperative chest tightness and chest pain may be considered for coronary angiography earlier. As confirmed by coronary angiography, ISR was defined as ≥50% luminal stenosis over the entire length of the stent and/or the 5-mm segment proximal and distal to the stent.

The median follow-up in this study was 386 days. During the perioperative period, a total of eight of the 414 patients developed arrhythmias (four sinus bradycardia, three second-degree atrioventricular block, and one incidental ventricular extrasystole), two had experienced mitral papillary muscle dysfunction, two had acute heart failure, and two had suffered from the postinfarction syndrome. All of these patients improved significantly after standardized treatment.

### Statistical analysis

**Figure 1:**
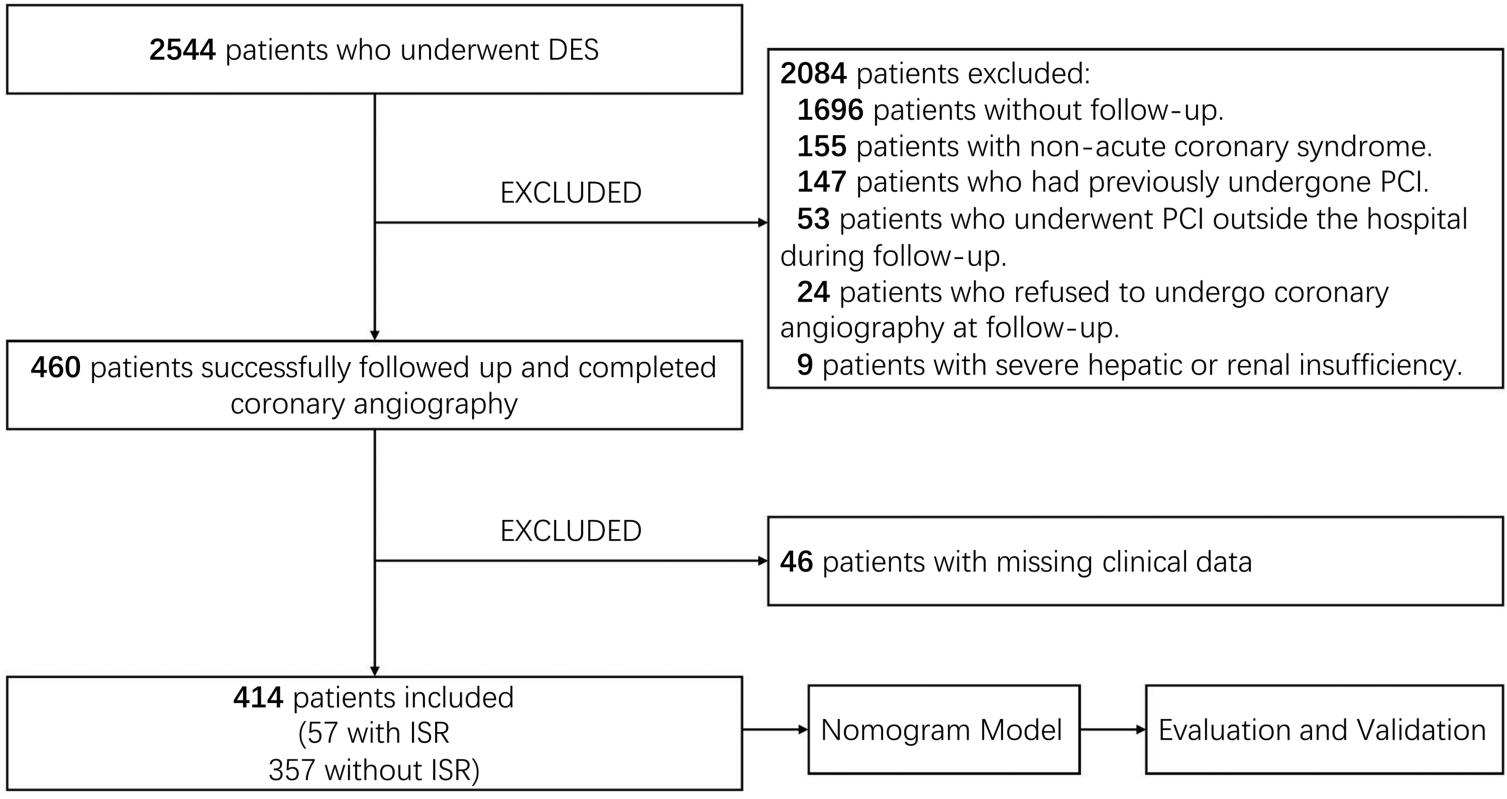
Flowchart of this study.

We used R 4.2.2 and SPSS 26.0 for data processing, curve drawing, and model building. Continuous variables were expressed as the mean ± standard deviation or median (p_25_, p_75_) according to normality, and categorical variables were expressed as the counts (%). LASSO regression analysis determined the best variable among the clinical variables. Then we constructed the nomogram prediction model using conditional multivariate logistic regression analysis based on the clinical variables selected in the LASSO regression analysis. To demonstrate the prediction accuracy of this nomogram model, we calculated the area under the curve (AUC) of the receiver operating characteristic (ROC) to evaluate its discriminant ability. In general, the diagnostic accuracy of AUC ≥ 0.6 can be considered and accepted (11). The calibration plots were used to compare the actual predictive power of the model with the ideal predictive power, and a small difference indicates good consistency of the prediction model. Moreover, we can also assess the clinical applicability and validity of the model using the decision curve analysis (DCA) and clinical impact curve (CIC), respectively. Figure 1 shows the flow chart of our study.

## Results

### Baseline characteristics

As shown in Table 1, 414 patients were enrolled in this study, of which 57 developed ISR after DES, and the incidence of ISR was 13.8% (57/414). We divided the 414 patients into the N-ISR group (317 patients) and the ISR group (57 patients). By comparison, it can be found that the significant differences in hypertension, diabetes mellitus, hyperuricemia, carotid plaque, mean stent diameter, number of stenosed vessels, stent number, total stent length, indirect bilirubin (IBIL), total bilirubin (TBIL), HbA1c, fibrinogen, and thyroxine between the two groups.

**Table 1:** Baseline characteristics of the study population.

	N-ISR (357)	ISR (57)	*P*-value
**Clinical Information**			
Gender			0.833
Male	243 (68.1%)	38 (66.77%)	
Female	114 (31.9%)	19 (33.3%)	
Smoking	123 (34.5%)	24 (42.1%)	0.262
Drinking	72 (20.2%)	11 (19.3%)	0.879
Age (years old)	64.00 (55.00,71.00)	67.00 (61.00,73.00)	0.159
BMI (kg/m^2^)	24.54 (22.43,26.61)	24.39 (22.05,26.49)	0.609
**Past Medical History**			
Hypertension	215 (60.2%)	43 (75.4%)	0.028
Diabetes mellitus	87 (24.4%)	22 (38.6%)	0.024
Hyperuricemia	27 (7.6%)	10 (17.5%)	0.014
Stroke	27 (7.6%)	8 (14.0%)	0.103
Respiratory diseases	46 (12.9%)	8 (14.0%)	0.811
Carotid plaque	155 (43.4%)	33 (57.9%)	0.041
Arrhythmia	37 (10.4%)	8 (14.0%)	0.408
**Medication History**			
ACEI/ARB	161 (45.1%)	28 (49.1%)	0.571
β-blocker	187 (52.4%)	28 (49.1%)	0.648
**Coronary Angiography**			
Number of stenosed vessel	2.00 (1.00,2.00)	2.00 (1.00,3.00)	0.008
Stent number	2.00 (1.00,2.50)	3.00 (2.00,4.50)	<0.001
Total stent length (mm)	41.00 (24.00,64.00)	70.00 (48.50,106.50)	<0.001
Mean stent diameter			0.015
≤2.83 mm	128 (35.9%)	30 (52.6%)	
>2.83 mm	229 (64.1%)	27 (47.4%)	
**Laboratory Findings**			
WBC (×10^9^/L)	6.70 (5.65,8.20)	6.10 (5.55,7.80)	0.227
RBC (×10^12^/L)	4.20 ± 0.53	4.08 ± 0.63	0.125
Hb (g/L)	131.00 (120.00,141.00)	127.00 (113.50,137.00)	0.051
HCT (%)	38.30 (35.20,41.05)	37.50 (32.95,40.45)	0.153
PLT (×10^9^/L)	184.00 (154.00,221.00)	191.00 (152.50,235.00)	0.483
N (×10^9^/L)	4.50 (3.60,5.70)	4.20 (3.50,5.35)	0.094
L (×10^9^/L)	1.40 (1.10,1.80)	1.50 (1.00,1.80)	0.863
AST (U/L)	23.00 (19.00,29.00)	22.00 (18.50,28.00)	0.459
ALT (U/L)	20.00 (15.00,29.00)	19.00 (13.00,25.50)	0.290
Total cholesterol (mmol/L)	3.59 (3.03,4.45)	3.81 (2.94,5.12)	0.511
Triglyceride (mmol/L)	1.37 (0.99,1.91)	1.39 (0.88,2.12)	0.932
HLD (mmol/L)	1.07 (0.92,1.25)	1.10 (0.88,1.37)	0.564
LDL (mmol/L)	1.83 (1.43,2.47)	1.84 (1.40,2.65)	0.682
Lp-a (g/L)	1.18 (1.06,1.31)	1.17 (1.01,1.35)	0.932
Lp-b (g/L)	0.64 (0.53,0.83)	0.69 (0.56,0.85)	0.282
Cystatin C (mg/L)	0.99 (0.86,1.13)	1.02 (0.89,1.21)	0.217
Creatinine (µmol/L)	73.00 (62.00,86.00)	73.00 (62.00,88.50)	0.641
Uric Acid (µmol/L)	327.00 (269.50,383.50)	324.00 (280.00,390.00)	0.485
GFR (ml/min·1.73 m^2^)	92.00 (80.00,106.00)	88.00 (75.00,105.00)	0.348
Homocysteine (µmol/L)	12.90 (10.70,16.30)	14.30 (10.30,18.20)	0.260
TBIL (µmol/L)	11.30 (8.80,14.85)	10.20 (7.35,13.40)	0.022
DBIL (µmol/L)	3.60 (2.70,5.00)	3.20 (2.50,4.50)	0.237
IBIL (µmol/L)	7.70 (5.90,9.95)	6.90 (4.70,8.45)	0.009
Albumin (g/L)	39.10 (36.90,40.80)	39.10 (35.65,41.75)	0.604
CRP (mg/L)	1.20 (0.50,3.45)	1.60 (0.65,5.20)	0.286
FBG (mmol/L	4.88 (4.49,5.69)	4.85 (4.43,6.13)	0.640
HbA1c (%)	6.00 (5.70,6.70)	6.40 (5.85,7.76)	0.001
INR	0.98 (0.94,1.04)	1.00 (0.96,1.07)	0.055
APTT (s)	26.90 (25.05,28.50)	27.60 (25.90,29.30)	0.059
Fibrinogen (g/L)	2.69 (2.33,3.12)	3.10 (2.44,3.84)	0.001
D-dimer (mg/L)	0.25 (0.18,0.41)	0.26 (0.16,0.51)	0.950
TSH (mIU/L)	1.63 (1.06,2.52)	1.79 (1.15,2.58)	0.670
Thyroxine (nmol/L)	93.80 (83.87,106.79)	104.83 (87.46,120.39)	0.003
**Imaging Findings**			
LVEF (%)	65.10 (61.00,68.70)	66.00 (59.85,69.90)	0.665
LAD (mm)	33.00 (30.00,36.00)	32.00 (29.00,36.00)	0.342
LVDs (mm)	30.00(28.00,33.10)	29.50(27.90,33.00)	0.443
LVDd (mm)	47.35 ± 4.93	47.36 ± 4.81	0.982

WBC, white blood cell; RBC, red blood cell; Hb, hemoglobin; HCT, hematocrit; PLT, platelet; N, neutrophil; L, lymphocyte; AST, aspartate aminotransferase; ALT, alanine transaminase; LDL, low density lipoprotein; Lp-a, lipoprotein-a; Lp-b, lipoprotein-b; GFR, glomerular filtration rate; TBIL, total bilirubin; DBIL, direct bilirubin; IBIL, indirect bilirubin; CRP, C-reactive protein; FBG, fasting blood-glucose; INR, international normalized ratio; APTT, activated partial thromboplastin time; TSH, thyroid-stimulating hormone; LVEF, left ventricular ejection fraction; LAD, left atrial diameter; LVDs; left ventricular systolic diameter; LVDd, left ventricular diastolic diameter.

### Correlation between variables

**Figure 2:**
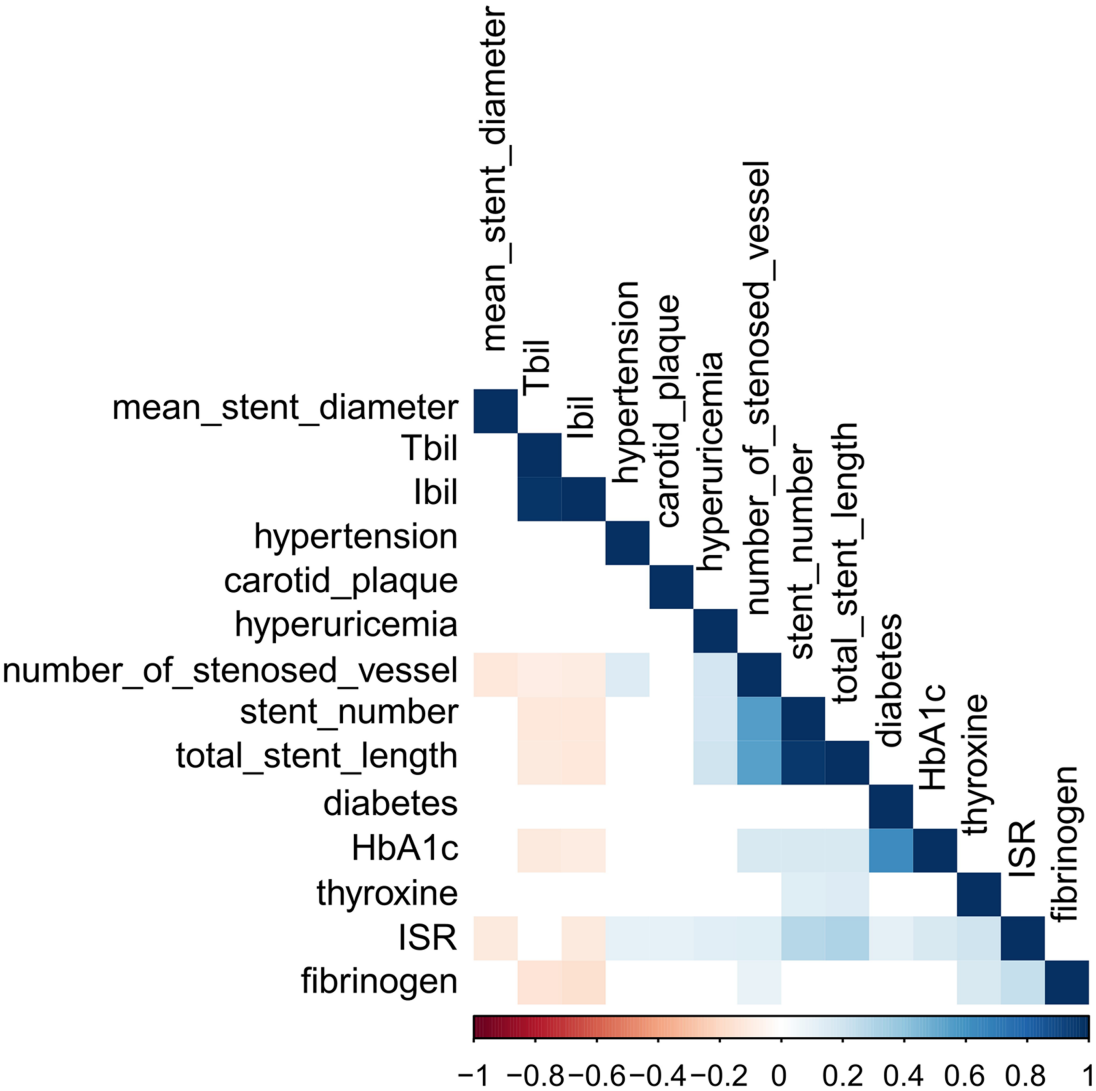
Correlation analysis between variables.

We performed multivariate correlation analysis for variables that were statistically significant in the baseline data, and the results are shown in Figure 2, in which we can observe a certain correlation between variables and ISR as well as between different variables (blue represents positive correlation, red represents negative correlation, and white represents no correlation). Interestingly, we noted that IBIL and mean stent diameter, unlike other clinical parameters, were negatively correlated with the occurrence of ISR.

### LASSO regression analysis

**Figure 3:**
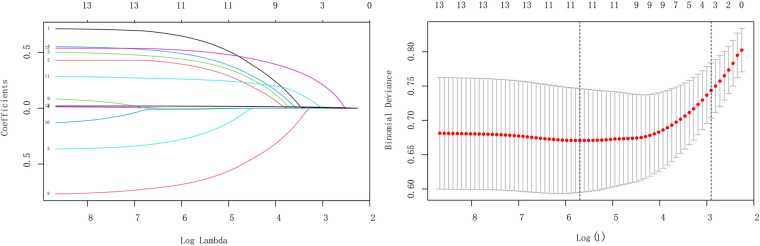
LASSO regression analysis of clinical variables.

LASSO regression could impose a regression penalty on all variable coefficients such that relatively unimportant independent variable coefficients become 0 and are thus excluded from the model. The main difference between LASSO regression and traditional stepwise regression is that it can process all independent variables simultaneously instead of stepwise, and this improvement greatly increases the stability of the modeling. Due to the large number of research variables included in this study and the correlation between different variables, We used LASSO regression to screen the variables to prevent the model from overfitting and to select the characteristic variables that predict the risk of ISR. In this study, the lambda-min was taken as the optimal value of the model to screen the best variable, and we counted the variables with non-zero regression coefficients. The results of LASSO regression analysis showed that hypertension, diabetes mellitus, hyperuricemia, carotid plaque, mean stent diameter, number of stenosed vessels, total stent length, IBIL, HbA1c, fibrinogen, and thyroxine were predictive factors for ISR in patients with CHD after DES (Figure 3).

### Multivariate logistic regression analysis

Furthermore, we included hypertension, diabetes mellitus, hyperuricemia, carotid plaque, mean stent diameter, number of stenosed vessels, total stent length, IBIL, HbA1c, fibrinogen, and thyroxine in multivariate forward stepwise logistic regression analysis. The results showed that mean stent diameter (OR = 0.481, 95%CI = 0.255–0.909, *P* = 0.024), total stent length (OR = 1.017, 95%CI = 1.010–1.025, *P* < 0.001), HbA1c (OR = 1.436, 95%CI = 1.094–1.884, *P* = 0.009), fibrinogen (OR = 1.712, 95%CI = 1.287–2.278, *P* < 0.001) and thyroxine (OR = 1.020, 95%CI = 1.006–1.035, *P* = 0.005) were all independent risk factors affecting the occurrence of ISR in patients with CHD after DES (Table 2). Although hypertension was not statistically significant (*P* = 0.051) in the multivariate logistic regression analysis, we still included hypertension obtained by LASSO regression analysis in the model considering the clinical practicability of LASSO regression analysis, the two-sided statistical significance level and the conclusions of previous studies (12–14).

**Table 2:** Multivariate logistic regression analysis of predictors.

Predictors	β value	OR	95%CI	*P*-value
Hypertension	0.709	2.032	0.996–4.145	0.051
Mean stent diameter	−0.732	0.481	0.255–0.909	0.024
Total stent length	0.017	1.017	1.010–1.025	<0.001
HbA1c	0.362	1.436	1.094–1.884	0.009
Fibrinogen	0.538	1.712	1.287–2.278	<0.001
Thyroxine	0.020	1.020	1.006–1.035	0.005

**Figure 4:**
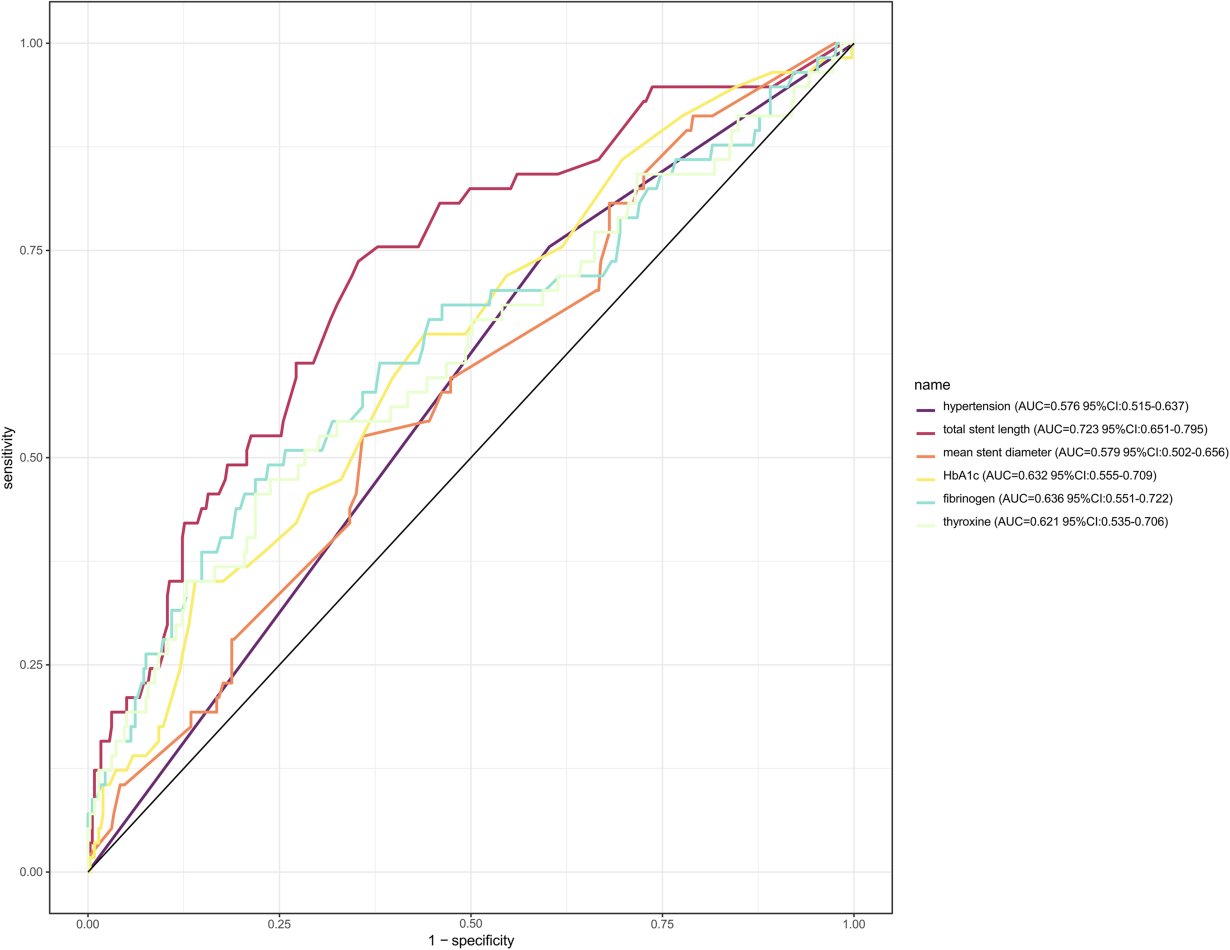
ROC curve and the diagnostic performance of individual predictors.

And the ROC curves of each variable were plotted separately. As shown in Figure 4, the total stent length had the largest AUC value for predicting the risk of ISR, with an AUC value of 0.723 (95%CI = 0.651–0.795), followed by 0.636 (95%CI = 0.551–0.722) for fibrinogen.

### Interaction analysis

When the influence of a predictor on the dependent variable varies with the levels of other predictors, it suggests the presence of an interaction effect between predictors. The presence of interaction effects indicates that the effects of multiple factors examined simultaneously are not independent. It measures the degree to which the effects of different levels of one factor depend on the levels of another or multiple factors. Many studies have neglected the potential impact of interaction effects on outcome events (5, 7, 8, 15). In this study, interaction analysis of the selected predictors was performed based on the results of multivariate logistic stepwise regression analysis. Table 3 shows no interaction effect between different variables on the occurrence of ISR after DES (*P* > 0.05). Therefore, we excluded the potential influence of interaction effects between variables on the model, which further improved the model's reliability.

**Table 3:** Interaction analysis of predictors.

Predictors	Hypertension	Total stent length	Mean stent diameter	HbA1c	Fibrinogen	Thyroxine
Hypertension	NA	0.0651	0.7428	0.8024	0.7499	0.4833
Total stent length	0.0651	NA	0.7711	0.2529	0.3627	0.8550
Mean stent diameter	0.7428	0.7711	NA	0.8537	0.1053	0.7557
HbA1c	0.8024	0.2529	0.8537	NA	0.3636	0.1053
Fibrinogen	0.7499	0.3627	0.1053	0.3636	NA	0.2831
Thyroxine	0.4833	0.8550	0.7557	0.1053	0.2831	NA

### Construction of nomogram prediction model

**Figure 5:**
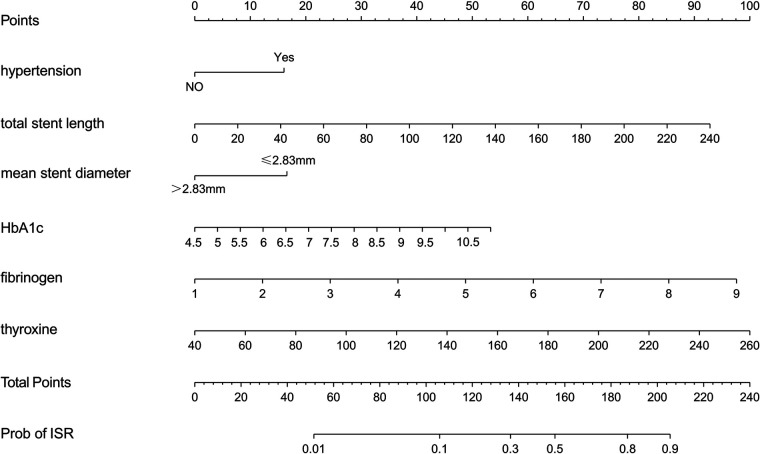
Nomogram to predict the probability of ISR.

We used these six variables as predictors to establish a nomogram prediction model for ISR after DES (Figure 5). The interpretation method of the nomogram was as follows: a vertical line was drawn on the horizontal axis of each predictor, corresponding to a specific score on the horizontal axis of “Points”; the scores corresponding to the six predictive factors were added to obtain the total score, and the value on the horizontal axis of “Prob of ISR” corresponding to the total score was the risk prediction value of the patient.

### Evaluation and validation of the nomogram model

**Figure 6:**
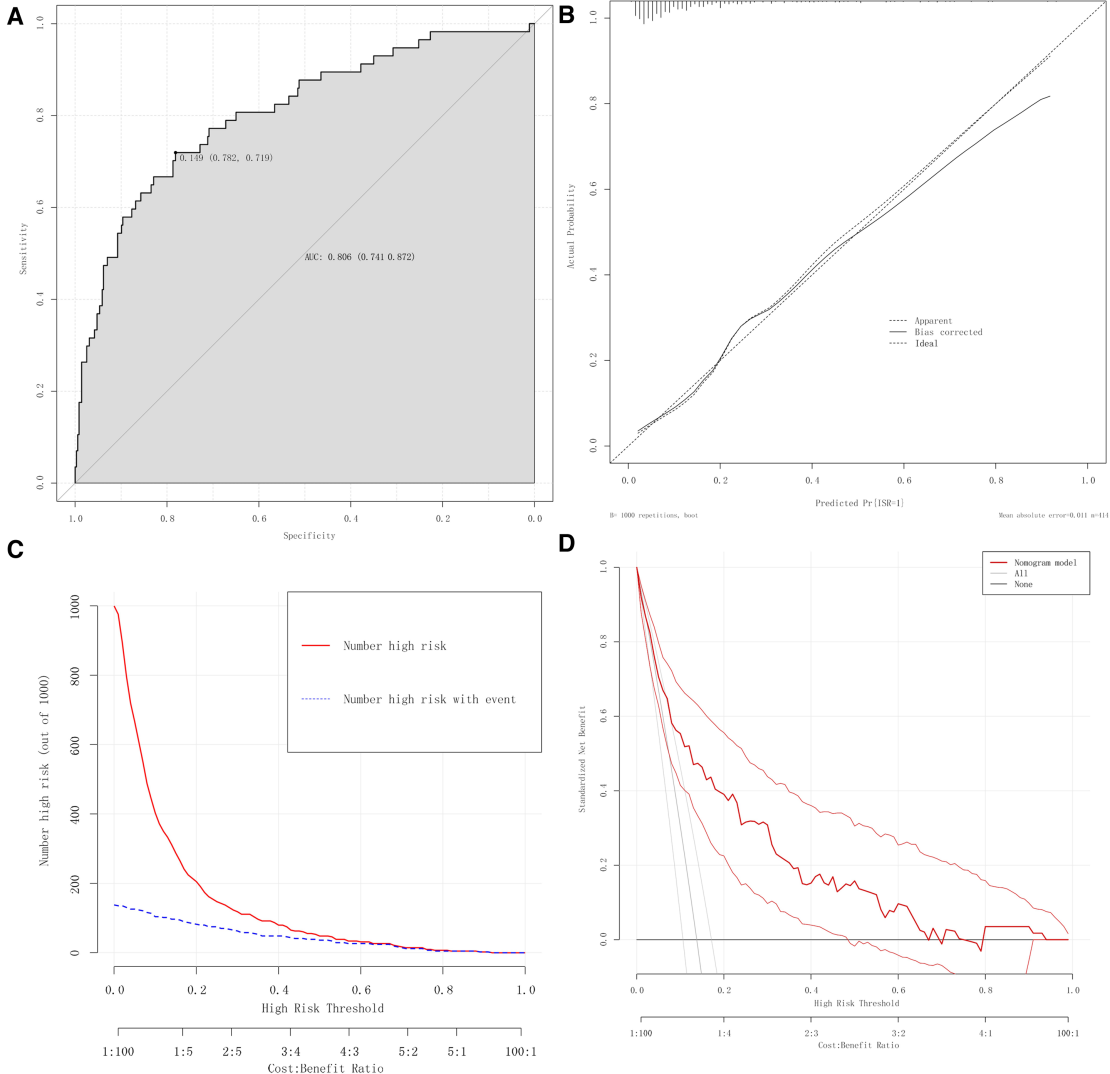
Evaluation of the nomogram prediction model. **(A)** ROC curves of the nomogram. **(B)** Calibration plots of the nomogram. **(C)** CIC of the nomogram. **(D)** DCA of the nomogram.

This study evaluated the nomogram prediction model for ISR in terms of the model's discrimination, calibration, validity, and clinical practicality. The AUC value of the nomogram was 0.806 (95%CI: 0.739–0.873), which was larger than the AUC value of any single predictor in Figure 4 for predicting ISR, indicating that the discrimination power of the nomogram model was good (Figure 6A).

We used calibration plots to assess the consistency of the model, where the abscissa represents the predicted risk of ISR, and the ordinate represents the actual risk of ISR. In this study, the high-quality calibration plots showed that the nomogram prediction model has strong consistency compared to the ideal model, indicating that there is no significant deviation between the predicted probability and the actual probability (Figure 6B).

The nomogram prediction model was analyzed using a clinical impact curve (CIC). The horizontal axis is the threshold probability, and the vertical axis is the number of people. The red curve represents the number of people predicted to be at high risk at different threshold probabilities, and the blue curve represents the number of people predicted to be at high risk by the model and the actual outcome events occurring at different threshold probabilities. When the threshold probability is greater than 50%, the population at high risk for ISR identified by this prediction model is highly consistent with the actual population with ISR, confirming the high clinical efficiency of this prediction model (Figure 6A).

Moreover, with the occurrence of ISR as the state variable and the risk prediction value as the test variable, the DCA curve suggested that the level of net clinical benefit to patients was high and that this model had good clinical applicability (Figure 6B).

The conventional method of dividing the entire sample into training sets and validation sets may lead to the accidental selection or omission of some variables due to the uncertainty of the random grouping, which would ultimately affect the stability and authenticity of the model. This effect is pronounced in the data sets with small sample sizes, and many researchers have ignored this point. We adopted the method of bootstrap verification combined with 10-fold cross-validation. In the process of multiple sampling, the model could be repeatedly verified, and the results could better prove the stability and reliability of the model. Bootstrap verification is performed by randomly drawing samples from the original dataset with the same number of samples as the size of the original queue. In our study, the cohort obtained by bootstrap sampling also included 414 patients, with each having the same probability of being sampled. Each bootstrap sampling result typically includes at least about two-thirds of the patients in the original cohort. This process is repeated 1,000 times to generate 1,000 model performance metrics. The corrected C index was calculated to be 0.804 (95%CI: 0.802–0.806). The 10-fold cross-validation method randomly divides the original data into ten groups, alternately uses nine groups to build the model and the remaining one group of data to validate the model, and then calculates the average of the ten results. We repeated this procedure 1,000 times and calculated a corrected C-index of 0.787 (95%CI: 0.780–0.795).

### The predictive value of thyroxine for ISR

We constructed a nested model without thyroxine. Analysis of variance was used to compare the goodness of fit between the nomogram prediction model and the nested model. The results showed that the fitting effect of the two models was significantly different (F = 9.179, *P* = 0.003), and the predictive effect of the nested model without thyroxine was weaker than that of the nomogram prediction model. We then calculated the AIC values for the two models separately. It was found that the AIC value of the nested model without thyroxine was 231.62, while the AIC value of the nomogram prediction model was 224.39, indicating that the model with thyroxine was better than that without thyroxine.

The net reclassification index (NRI) is an indicator to compare the predictive ability of two models. Compared to AUC and other indicators, NRI has higher sensitivity and better clinical interpretation. When NRI > 0, the new model is better at predicting the event than the old model. When NRI < 0, it indicates that the predictive power of the new model is decreasing. In this study, less than 30% of the predicted risk of the model was considered a low-risk group, and more than 70% was considered a high-risk group. We calculated the NRI values for the nomogram prediction model and the nested mode, and the results showed that NRI = 0.134 (95%CI: 0.0031–0.2649, *P* = 0.044), indicating that the predictive ability of the nomogram model including thyroxine was improved compared with the nested model, and the proportion of correct classification was increased by 13.4%.

Our previous study has found that thyroxine could be used as an independent predictor of ISR after PCI (OR = 1.020, 95%CI: 1.006–1.035, *P* = 0.005). The ROC curve also showed that thyroxine had a good discriminative ability (AUC = 0.621). Combined with ANOVA, as well as AIC and NRI values, it is further confirmed that thyroxine has a predictive potential for the occurrence of ISR after PCI, which provides us with a new perspective in future studies of ISR.

## Discussion

PCI is an essential method for the treatment of coronary heart disease. Compared to BMS, the use of DES technology has dramatically improved the efficacy and safety of PCI. However, ISR and the demand for target lesion revascularization still occur at a rate of 1%–2% per year, making the prevention and treatment of ISR a complex problem in the cardiovascular field (16). Compared with PCI for new lesions, PCI for ISR has accounted for approximately 10% of all PCI in the United States over the past decade and has been associated with a higher risk of major adverse cardiac events (17, 18).

The exact mechanism of ISR formation remains unclear. Therefore, early identification and control of ISR-related risk factors may be an important method to reduce the incidence of ISR. Establishing an effective prediction model may provide a helpful reference for the prevention of ISR. Nomogram is currently widely used in the medical field to establish predictive models. By integrating various prognostic events and crucial variables, the nomogram can generate individual probabilities of clinical events, which satisfies our desire for clinical models and promotes the progress of personalized medicine. Compared with traditional prediction models, such as heat maps or scoring systems, the advantages of nomograms are that they are easy to use and understand. There is no need to convert continuous variables into categorical variables, and the length of the lines in the nomogram can judge the relative importance of predictor variables. With a user-friendly digital interface for rapid calculation, the nomogram model improves predictive accuracy and is easier to use than traditional models, which helps us make clinical decisions promptly and effectively (19).

In this study, we retrospectively analyzed the clinical characteristics of 414 CHD patients who underwent first-time PCI with DES and investigated the incidence of ISR in these patients and the predictors of ISR. Firstly, we found that the incidence of restenosis after DES was 13.8%. Secondly, hypertension, mean stent diameter, total stent length, HbA1c, fibrinogen, and thyroxine were independent predictors of ISR. And the nomogram prediction model, including these predictors, has a good predictive value for the occurrence of ISR. Finally, we further explored the predictive value of thyroxine in ISR after PCI.

The effect of hypertension on ISR has been confirmed in many studies. The primary mechanism is that hypertension increases the impact of blood flow on the vessels, causing damage to vascular endothelial cells, resulting in the formation of atherosclerotic plaques, and ultimately increasing the risk of ISR (20). Zhao et al. reviewed comprehensive data on 398 patients with CHD undergoing percutaneous coronary intervention plus SES. They found that hypertension can be used as an independent predictor of increased risk of ISR (21). The findings of Sajadian et al. also suggest that hypertension and diabetes are the most probable factors affecting ISR (13). HbA1c reflects glycemic control over the last 2 to 3 months. Several studies have shown that elevated HbA1c level is an independent predictor of poor prognosis after PCI (22). Karadeniz et al. showed that a higher HbA1c level was associated with a higher incidence of ISR in diabetic patients with STEMI undergoing primary PCI (23). We speculate that the possible mechanism of ISR caused by HbA1c is that the continuous increase of HbA1c increases the viscosity of red blood cells. High blood viscosity may cause damage to vascular endothelial cells, enhance the release of endothelin and reduce the release of nitric oxide and prostacyclin, thereby impairing vasomotor function. The continuous increase in HbA1c may also lead to the aggravation of protein glycosylation and oxidation, and advanced glycation end-products may promote the development of atherosclerosis. Our study confirms that elevated HbA1c level is an independent predictor of ISR after PCI and supports previous studies' conclusion that poor glycemic control in diabetes is the main cause of ISR after PCI (24, 25).

Previous studies have suggested that stent length and diameter appear to be important factors in the occurrence of ISR. Dietz et al. found a significant reduction in the occurrence of ISR in patients with a mean stent length of approximately 9 mm compared to patients with a mean stent length of approximately 16 mm (26). The study by Nita et al. discovered that patients with ISR had smaller stent diameters and longer stent lengths than controls (27). Moreover, Zhou et al. found that a smaller minimum stent diameter was associated with the incidence of ISR within three years after DES implantation in elderly ACS patients (8). Our study also confirmed these views. We believe that PCI is an invasive procedure with certain mechanical stimulation, which may cause vascular endothelial cell damage, increase the risk of thrombosis, stimulate the proliferation and migration of smooth muscle cells, and lead to luminal proliferative stenosis. Therefore, the longer the stent length, the more severe the vascular endothelial damage. In clinical practice, we should choose the appropriate stent size for patients with CHD to reduce the occurrence of ISR.

Fibrinogen is the precursor of fibrin, which is involved in the process of inflammation and thrombosis. Elevated fibrinogen levels are a recognized risk factor for adverse cardiovascular events in patients with coronary heart disease (28, 29). Many studies have shown that fibrinogen levels are associated with the occurrence of ISR after PCI (30–32). Our study also confirmed that fibrinogen is an independent predictor of ISR. Chai et al. suggested that fibrinogen and its metabolites can stimulate endothelial cell degeneration, increase the release of endothelial cell-derived growth factors, lead to endothelial dysfunction, and stimulate the growth of smooth muscle cells, ultimately leading to the occurrence of ISR (32).

There is currently a great deal of controversy regarding the effect of thyroid hormones on CHD. Most studies believe that high levels of thyroid hormones could increase the risk of CHD and its complications. In contrast, some studies believe that low levels of thyroid hormones might be considered a risk factor for CHD (33). Even some studies reported no significant correlation between them (34). Bano et al. conducted a large prospective cohort study, the Rotterdam Cohort Study, to investigate the relationship between thyroid function and atherosclerosis. They found that FT4 levels were associated with coronary artery calcification and positively and linearly associated with atherosclerotic cardiovascular disease (ASCVD) events and mortality. They suggested that FT4 should be considered a new predictor of ASCVD risk (35). Jung and Cols also reported that serum FT4 levels in 192 patients with stable angina pectoris were higher even within the normal reference range and significantly correlated with the presence and severity of coronary artery disease (36). Studies have shown that thyroid hormones may directly or indirectly affect the formation of atherosclerosis by altering vascular tone, regulating macrophage function, promoting angiogenesis, and regulating vascular smooth muscle cell proliferation. Attabak Toofani Milani et al. demonstrated a potent effect of thyroid hormones on gene and protein expression levels of major mediators of pro-inflammatory, angiogenic, and endothelial dysfunction associated with the development of atherosclerosis (37).

However, there are few studies on the effect of high levels of thyroxine on ISR after PCI. Only Canpolat et al. found that high preoperative serum FT4 level was an effective independent predictor of BMS restenosis in patients with stable and unstable angina pectoris. They suggested that serum FT4 increases the risk of ISR by enhancing the activity of the renin-angiotensin system and the proliferation of vascular smooth muscle cells (38). Therefore, this study is the first to identify thyroxine as a significant predictor of ISR after drug-eluting stent implantation. Moreover, we further confirmed the high predictive value of thyroxine for ISR by a series of other means.

In summary, we have developed an ISR prediction nomogram model based on hypertension, mean stent diameter, total stent length, HbA1c, fibrinogen, and thyroxine, which was evaluated and validated to help clinicians identify high-risk ISR patients and optimize treatment strategies, thereby improving the prognosis of these patients.

## Limitation

Inevitably, there are some limitations to this study. (i) This is a retrospective study, and the data of this study are only from the Fourth Affiliated Hospital of Zhejiang University School of Medicine, with small sample size and single sample source, which may affect the accuracy of the study results. Therefore, the conclusions still need to be verified by further prospective multicenter cohort studies with large sample sizes. (ii) Only internal validation was performed in this study, which made the extrapolation of the nomogram prediction model still unknown. For external validation, it is still necessary to select patients with coronary heart disease after DES from other medical centers. (iii) We have confirmed that thyroxine can be used as an important predictor of ISR, but further clarification of the effect of thyroxine on ISR under different conditions, such as hyperthyroidism, hypothyroidism, and normal levels, will help us to further formulate precise interventions. (iv) We analyzed the impact of stent characteristics on ISR while ignoring the impact of the characteristics of the culprit blood vessels on ISR. This will be the focus of our subsequent study.

## Conclusion

Hypertension, mean stent diameter, total stent length, HbA1c, fibrinogen, and thyroxine are important predictors of ISR. We developed and validated a personalized nomogram model to predict the risk of ISR after PCI in patients with CHD. It is based on these six common and easily accessible clinical indicators, providing clinicians with a simple and practical assessment tool. Moreover, this nomogram model has good accuracy, which can better identify the high-risk population of ISR and provide practical decision-making information for the follow-up intervention of the high-risk population.

## Data Availability

The raw data supporting the conclusions of this article will be made available by the authors, without undue reservation.
